# The impacts of improvements in the unified economic and environmental efficiency of transportation infrastructure on industrial structure transformation and upgrade from the perspective of resource factors

**DOI:** 10.1371/journal.pone.0278722

**Published:** 2022-12-09

**Authors:** Yijiao Wang

**Affiliations:** School of Business, Xi’an University of Finance and Economics, Xi’an, Shaanxi, China; East China University of Science and Technology, CHINA

## Abstract

As an important part of a modern economic system, a modern industrial system is the key to promoting high-quality economic development. China’s modern industrial system construction focuses on industrial restructuring. At present, in order to strengthen the support and leading role of transportation in the modern economic system, China is actively promoting the construction of a competitive transportation power. Therefore, it is necessary to study whether large-scale investment in transportation infrastructure can promote industrial structure transformation and upgrade. This paper takes China as the research background. Firstly, a RAM model was employed to evaluate the unified economic and environmental efficiency of transportation infrastructure that measures the level of transportation infrastructure investment. Secondly, a PVAR model was built to evaluate the dynamic effects of transportation infrastructure investment on industrial structure transformation and upgrade. Finally, from the perspective of rational flow and optimal allocation of resource factors, the paper points out that transportation infrastructure investment can indirectly promote industrial structure transformation and upgrade through three paths, namely expanding market demand, reducing resource misallocation and increasing technological innovation, and the first half of these paths are positively regulated by policies. Then, an empirical test was done with the moderated mediation model. Research findings suggest that: first, improvements in the unified economic and environmental efficiency of transportation infrastructure can only promote industrial structure supererogation in a short time, but have no significant effect on promoting industrial structure rationalization in the short or long term. Second, in actual situation, transportation infrastructure investment can promote industrial structure transformation and upgrade only by expanding market demand and technological innovation, but not by reducing resource misallocation. Third, the first half of these paths through which transportation infrastructure investment promotes industrial structure transformation and upgrade are positively regulated by policies. This paper provided some theoretical reference for promoting industrial structure transformation and upgrade by virtue of the sustainable development of transportation.

## 1. Introduction

After decades of construction, great progress has been made in China’s transportation infrastructure. China is now leading the world in the scale of transportation infrastructure. According to the *Statistical Communique on Transportation Development 2019* [1], by the end of 2019, the total length of China’s comprehensive transportation network had exceeded 5 million kilometers, including 149,600 kilometers of expressways and 35,000 kilometers of high-speed railways, 2,520 10,000-ton port berths, and more than 6,700 kilometers of urban rail transit in operation, ranking first in the world. With the continuous progress in transportation reform, China is striding from a large transportation power to a competitive transportation power. Industrial structure is an important economic indicator. After the reform and opening up, China first focused on the secondary sector and implemented an industrial structure dominated by the secondary sector and balanced by the primary and tertiary sectors, leading to rapid expansion of the economy. However, the value added of the secondary sector began to decline after reaching a peak of 47.95% of GDP in 2006. Since around 2013, the conditions and environment for industrial development in China have undergone profound changes, and the industrial structure has also undergone major restructurings. The tertiary sector has become the core contributor to economic growth. According to the changes in and characteristics of economic development, China’s economy has shifted from a stage of high-speed growth to one of high-quality. As an important part of a modern economic system, a modern industrial system is the key to promoting high-quality economic development. China’s modern industrial system construction focuses on industrial restructuring. At present, China is actively promoting the construction of a competitive transportation power. Requirements for high-quality development are reflected in transportation. Moreover, transportation infrastructure as the vanguard of development can optimize industrial restructuring and promote high-quality economic development. Therefore, evaluating the efficiency of current transportation infrastructure investment and exploring its impacts on industrial structure transformation and upgrade can provide a reference and basis for formulating effective transportation infrastructure investment policies to guide industrial structuring and accelerate high-quality economic development.

Studies about the impacts of transportation infrastructure on industrial structuring started with the economic location theory. Hoover’s study shows that reasonable distribution of transportation infrastructure can facilitate effective agglomeration of enterprises and economic regions [2]; Taaffe et al. built transportation-region development models from a regional perspective to show the relationship between transportation infrastructure and regional industrial development [3]; Krugman proposed the famous core-periphery model to investigate whether transportation costs have significant impacts on industrial agglomeration [4]. Later, relevant studies show that impacts of transportation infrastructure on industrial structure transformation and upgrade are both direct and indirect. The direct impacts of transportation infrastructure on industrial structure transformation and upgrade are as follows: in the construction stage, transportation infrastructure can directly affect industrial structure transformation and upgrade by increasing regional capital inflows, employment and income; in the operation stage, transportation infrastructure can directly affect industrial structure transformation and upgrade by effectively reducing transportation costs, promoting factor mobility and reducing inventory levels. Studies by Jiwattanakulpaisarn and Zhang suggest that transportation infrastructure construction can increase employment and income, thus stimulating regional consumption of industrial goods and services and driving the development of industry and services [5, 6]; Selod and Soumahoro, Liu et al. suggest that the operation of transportation infrastructure can directly reduce production costs and inventory investment for enterprises, so that enterprises can have more funds to invest in R&D and innovation, making time-sensitive and value-added products go to market faster, thus boosting industrial development [7, 8]; the indirect impacts of transportation infrastructure on industrial structure transformation and upgrade are as follows: in the operation stage, transportation infrastructure can directly affect industrial structure transformation and upgrade by promoting income growth in poverty-stricken areas, international trade, foreign direct investment and technological innovation, etc. Studies by Estache, Fan and Zhang, Chen et al. suggest that for poor areas, the development of transportation infrastructure can effectively reduce poverty, increase non-agricultural employment opportunities to facilitate the transfer of labor to the secondary and tertiary industries, and help increase income by unclogging sales channels for local products [9–11]; studies by Bougheas et al., Francois et al. and Sun et al. suggest that the development of transportation infrastructure can significantly increase trade flows in both coastal and inland areas [12, 13]; studies by Chen and Chen suggest that improvements in transportation infrastructure can enhance regional attraction to foreign direct investment, and improve regional manufacturing structure through technology spillover, knowledge spillover, industrial migration and demonstration effect. However, different transportation infrastructures have different influence mechanisms and degrees [14]; Ma’s study suggests that the development of transportation infrastructure is helpful to break the barriers to the flow of innovation factors within the region, such as talents and capital, promote the spillover of tacit knowledge and technological innovation, which can transform traditional industries with new technologies and new equipment, thus speeding up industrial structure supererogation [15].

The heterogeneity of transportation infrastructure’s impact on industrial restructuring is also worthy of attention. Overall, transportation infrastructure is more conducive to promoting the development of services and high-tech manufacturing. Studies by Chandra and Thompson suggest that in the 1960s, development of highways in America led to the development of industry and services along the highways [16]; Holl’s study suggests that development of expressways in Spain improved regional accessibility and attracted manufacturers in large numbers to form clusters [17]; studies by Haines and Margo suggest that since the opening of railways in the mid-19th century in America, the value of agricultural production has decreased, but service-sector output has increased, while accessibility to regions along the railways has improved markedly [18]; studies by Sun et al., Wang and Wu suggest that the construction and improvement of transportation infrastructure can benefit the development of secondary and tertiary industries and promote industrial structure upgrading according to industrial differences [19, 20]. The impacts of transportation infrastructure development on cities along transportation infrastructure is heterogeneous. Studies by Mao and Wang conclude that the efficiency of accessibility between cities is not high, but there is an inverted u-shaped relationship between accessibility of central cities and urban industrial growth [21].

To sum up, first of all, the indicators of transportation infrastructure in the existing literature were mainly measured by the investment cost, construction quantity and structural layout, while the investment efficiency was rarely considered. As for the existing researches on the efficiency of transportation infrastructure investment, domestic and foreign scholars have conducted a large number of researches on the economic impact, environmental impact and social impact of transportation infrastructure investment respectively. However, most of these literatures were based on only one research dimension when analyzing the impact of certain transportation infrastructure investment. That is, studies were only conducted from one side of economic dimension, environmental dimension or social dimension, while few studies comprehensively considered the impact of transportation infrastructure input from two or three dimensions. This leaves some space for the research of this paper to expand. Secondly, due to the different production characteristics of various industries, the development of transportation infrastructure has a great difference in the impact of various industries. Therefore, existing studies have shown that transportation infrastructure has a heterogeneous impact on industrial structure adjustment. This provides reference for this paper to verify the relevant research conclusions. Finally, the existing literature discussed the mechanism of transportation infrastructure’s effect on industrial structure, which mainly promoted regional income, international trade, foreign direct investment and technological innovation. This provides a space for exploring other possible mechanisms in this paper.

The main research work and achievements of this paper are as follows: First, this paper studied the indicators of transportation infrastructure, from the efficiency angle, and based on the concept of sustainable development, through the input-output ratio between transportation infrastructure investment and integrated economic and environmental development. That is, the unified economic and environmental efficiency of transportation infrastructure was used, at the same time by using the RAM model to measure transportation infrastructure investment level. Secondly, panel vector autoregression model was used to investigate whether the unified economic and environmental efficiency of transportation infrastructure could promote the transformation and upgrading of industrial structure. Thirdly, from the perspective of reasonable flow and optimal allocation of resource elements, this paper put forward the specific mechanism of the effect of the unified economic and environmental efficiency of transportation infrastructure on the transformation and upgrading of industrial structure. Compared with the existing related research, the contribution of this paper may place mainly embodied in the following several aspects: first, this article was based on the sustainable development measures of transportation infrastructure investment level compared to the traditional measures would be more reasonable, to this stage of the real transportation infrastructure investment levels for a more comprehensive and accurate evaluation. Secondly, the econometric model was used to investigate the relationship between the unified economic and environmental efficiency of transportation infrastructure on the transformation and upgrading of industrial structure in the short and long term from a dynamic perspective, which has a certain validation and complementary role for the existing research conclusions on the impact of transportation infrastructure on industrial structure adjustment. Thirdly, this paper provided a theoretical framework for exploring the effect mechanism of the unified economic and environmental efficiency of transportation infrastructure on the transformation and upgrading of industrial structure, and also put forward a quantitative analysis idea for testing whether there is difference between the effect mechanism and the reality.

## 2. Research assumptions

On the premise of effectively promoting the free flow and market-oriented allocation of resource factors, sound transportation infrastructure can accelerate the supply-side structural reform by virtue of market demand, market-oriented resource allocation and technological innovation, thus contributing to industrial structure transformation and upgrade. Therefore, this paper discusses the paths of transportation infrastructure investment promoting industrial structure transformation and upgrade from the perspective of rational flow and optimal allocation of resource factors.

The scale and structure of market demand have a certain impact on the industrial structure transformation and upgrade. From the perspective of the impact of market demand scale: Caves’ study shows that the growth rate of market demand is one of the most important factors to determine the industrial structure [22]. Fan’s study points out that the scale of market demand has an important impact on the implementation of national innovative infrastructure construction and the establishment of a micro innovation environment for industrial clusters to a certain extent [23]. From the perspective of the impact of market demand structure: studies by Malerba et al. reveal that the evolution of demand structure can effectively stimulate technological innovation in enterprises [24]. Domestic market demand is mainly influenced by income and income distribution, while foreign market demand is mainly influenced by export trade. The impacts of transportation infrastructure on market demand are mainly reflected in domestic economic development and export trade. The positive effect of transportation infrastructure construction on economic development has been widely recognized by scholars. In recent years, studies on the impacts of transportation infrastructure construction on import and export have gradually increased. Studies by Bougheas et al. based on the gravity model conclude that the increase in trade flows is inseparable from the strong support of sound transportation infrastructure [25]. As for the reason, Behrens’ study shows that whether trade flows increase depends on the cost of transportation [12]. Therefore, this paper puts forward Assumption 1:

H1: Transportation infrastructure investment can promote industrial structure transformation and upgrade by expanding market demand.

The extensive economic growth model that relies on large-scale investment of resource factors can no longer meet the requirements of rational allocation of resource factors for China’s high-quality economic development. Resource misallocation is largely caused by institutional soft environment factors and hard environment factors such as transportation infrastructure. Although current analysis is mainly focused on institutional factors, discussion on the factors influencing the transportation infrastructure construction has recently increased. Bu et al. included intermediate inputs in manufacturing into the scope of resource factors and found that China’s expressway construction has a significant impact on enterprise resource allocation [26]. Li et al. found that enhancing manufacturing resource allocation efficiency in non-central cities is inseparable from the high accessibility of expressways [27]. Ghani et al. explored the impact of the upgraded India’s Golden Quadrangle Highway Network has an impact on manufacturing-related activities and found that transportation infrastructure construction can indeed improve the efficiency of regional resource allocation [28]. Therefor, existing studies generally conclude that transportation infrastructure investment can help improve resource allocation efficiency at enterprise level, industry level and regional level. Resource allocation is one of the fundamental reasons for industrial structure transformation and upgrade. A few studies have confirmed that transportation infrastructure affects industrial structure transformation and upgrade by improving resource allocation efficiency. Li et al. found that high-speed railway plays a positive role in promoting the free flow of labor force and continuous accumulation of capital, which is conducive to regional industrial structure transformation and upgrade [29]. Therefore, this paper puts forward Assumption 2:

H2: Transportation infrastructure investment can promote industrial structure transformation and upgrade by reducing resource misallocation.

As China’s economic development enters a new normal, the focus of development has shifted from aggregate economy to stable growing economy with a symmetrical economic structure. The key to economic structure optimization lies in industrial structure optimization. Technological innovation is the core force to realize industrial structure transformation and upgrade. The existing literature extensively discusses how technological innovation affects industrial structure transformation and upgrade. Wu and Sun found that technological innovation plays a positive role in promoting industrial structure transformation and upgrade [30]. The mechanism is as follows: on the one hand, from the perspective of supply and demand, technological innovation has an impact on industrial input-output and resource allocation efficiency, thus contributing to industrial structure transformation and upgrade. From the perspective of supply, technological innovation can optimize the allocation of labor resources by promoting the effective division of labor and the free flow of labor between different industries. From the perspective of demand, pushed by demand, industries improve the quality of products and services to maintain the scale of output and obtain competitive advantages. Specifically, enterprises increase investment in technology research and development, which can effectively promote industrial structure transformation and upgrade. On the other hand, technological innovation forms synergy with other factors to promote industrial structure transformation and upgrade. According to studies by Ding et al., easing financing constraints on enterprises is conducive to technological innovation, and thus can effectively promote industrial structure transformation and upgrade [31]. Shi et al. found that strict environmental regulations can promote industrial structure transformation and upgrade by forcing technological innovation [32]. Transportation infrastructure for promoting effect of technology innovation embodies a concentrated reflection of the capital effect and the spillover effects [33], including capital effect performance in to invest in transportation infrastructure itself is a kind of capital accumulation process, to a exist in production function, the capital stock and physical capital accumulation contribute to the promotion of technological innovation [34]. The spillover effects are reflected in the following aspects: first, transportation infrastructure construction achieves regional accessibility and actively promotes the free flow of innovation factors between regions and extensive development of innovation activities on the premise of effectively reducing transport transaction costs [4]. Secondly, transportation infrastructure construction strengthens regional economic correlation and expands regional boundaries, which is conducive to accelerating the free flow of inter-regional competitive inputs such as resource factors and promoting the division of labor and agglomeration of industries, thus forming innovation [35]. Third, transportation infrastructure construction promotes the diffusion and exchange of shared input factors between regions, and strengthens the innovative learning effect on the basis of knowledge spillover [36]. To sum up, transportation infrastructure construction can positively promote technological innovation, and technological innovation can effectively promote the transformation and upgrading of industrial structure. Therefore, this paper puts forward Assumption 3:

H3: Transportation infrastructure investment can promote industrial structure transformation and upgrade through technological innovation.

The impacts of transportation infrastructure on market demand are mainly reflected in domestic economic development and export trade. Especially since the Belt and Road Initiative was put forward, improvements in transportation infrastructure have helped strengthen exchanges and cooperation in bilateral trade, thus becoming a priority and focus in the Belt and Road construction. It has positive impacts on domestic economic development and the expansion of foreign trade [37]. Therefore, this paper puts forward Assumption 4a:

H4a: The Belt and Road Initiative plays a positive moderating role between transportation infrastructure investment and market demand.

Studies by Lin et al. show that large-scale transportation infrastructure construction in developing countries can effectively reduce the possibility of resource misallocation among enterprises [38]. Studies by Luo et al. point out that large-scale transportation infrastructure construction can effectively reduce inequality in resource allocation among provinces and cities and narrow the urban-rural income gap [39]. Therefore, improvements in transportation infrastructure lay a certain material foundation for the free flow of production factors between regions and the rational allocation of resource factors. Especially since the Belt and Road Initiative was put forward, transportation infrastructure has been regarded as a priority. This has played a positive role in reducing the misallocation of existing resource and promoting the integrated, coordinated development of the economy along the routes. Therefore, this paper puts forward Assumption 4b:

H4b: The Belt and Road Initiative plays a positive moderating role between transportation infrastructure investment and resource misallocation reduction.

The increasingly perfect comprehensive transportation infrastructure network can effectively reduce transportation costs and improve accessibility. This is conducive to realizing the free flow of technological innovation factors and further forming trans-regional spillover of technological innovation [40]. Since the Belt and Road Initiative was put forward, the rapid development of transportation infrastructure has greatly promoted trade between the regions along the routes, facilitated the spillover of technological innovation in the regions along the routes, and enhanced the availability of technological innovation factors in less developed regions along the routes. Therefore, this paper puts forward Assumption 4c:

H4c: The Belt and Road Initiative plays a positive moderating role between transportation infrastructure investment and technological innovation.

**Fig 1:**
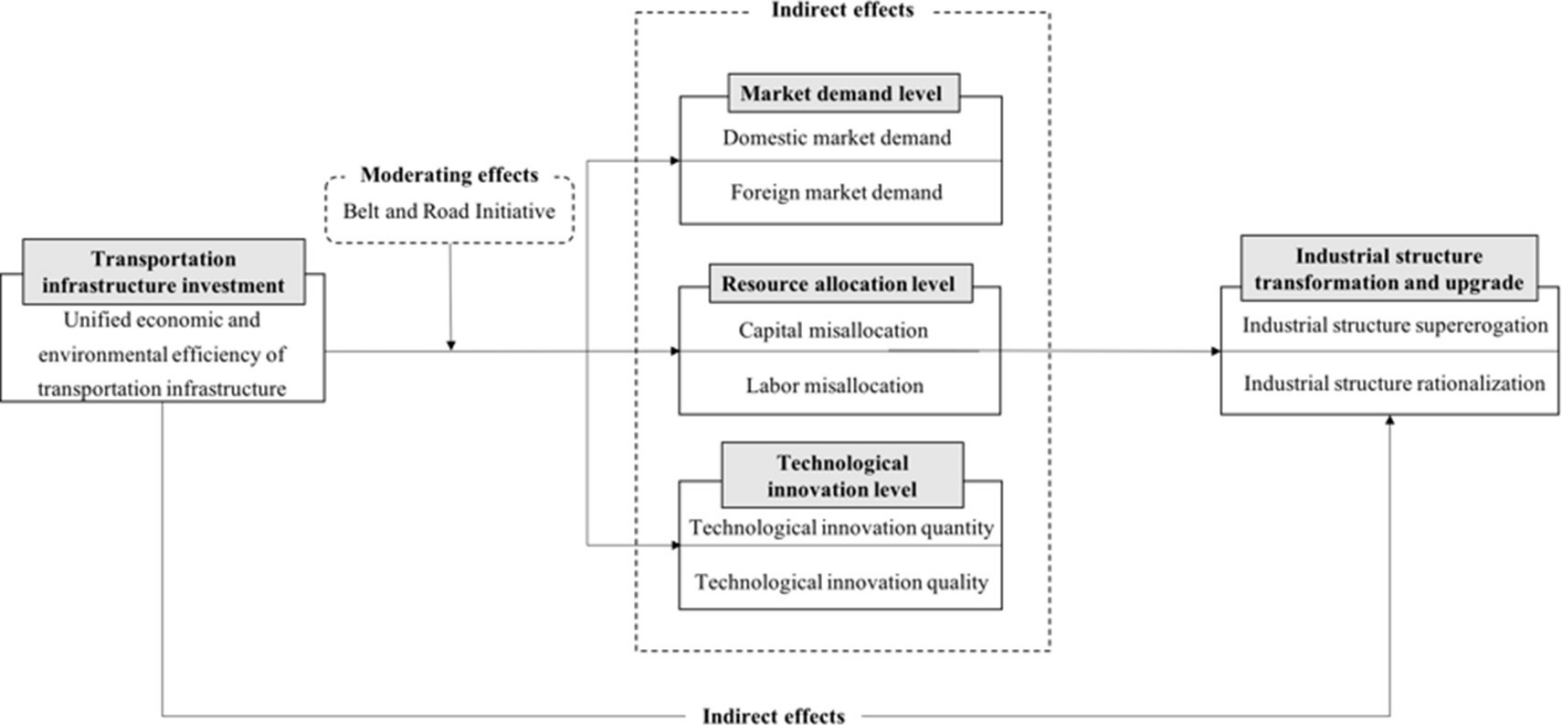
Mechanism of the unified economic and environmental efficiency of transportation infrastructure influencing industrial structure transformation and upgrade.

Based on the above mechanism analysis and research assumptions, this paper builds a mechanism framework of the unified economic and environmental efficiency of transportation infrastructure influencing industrial structure transformation and upgrade. The details are shown in Fig 1.

## 3. Methods

### 3.1. Range-adjusted measure (RAM) model

Before conducting the study, the unified economic and environmental efficiency of transportation infrastructure needs to be measured. The RAM model does not have issues such as “angle”, “radial” or non-slack variables in the traditional data envelopment analysis models, and so it can effectively measure the efficiency of decision-making units (DMUs) including the environment [41]. In addition, the RAM model has an additive structure, making it possible to measure separately economic efficiency and environmental efficiency and the unified efficiency of the two. Therefore, the RAM model was selected to measure the economic efficiency, environmental efficiency and unified efficiency of China’s transportation infrastructure in this paper.

#### 3.1.1. RAM model for unified efficiency

The additive structure of the RAM model is used to integrate the economic efficiency and the environmental efficiency in a single framework to obtain the RAM model for the unified efficiency:

$$max\left\{\begin{array}{c} (\sum_{n=1}^{N}R_{n}^{x}s_{n}^{x}+\sum_{m=1}^{M}R_{m}^{e}{(s}_{m}^{e^{+}}+s_{m}^{e^{-}})+\sum_{p=1}^{p}R_{p}^{y}s_{p}^{y}+\sum_{i=1}^{I}R_{i}^{b}s_{i}^{b})|\sum_{j=1}^{J}x_{nj}\lambda_{j}\\ +s_{n}^{x}=x_{nj},\forall n;\sum_{j=1}^{J}e_{mj}\lambda_{j}-s_{m}^{e^{+}}+s_{m}^{e^{-}}=e_{mj},\forall m;\sum_{j=1}^{J}y_{pj}\lambda_{j}-s_{p}^{y}=y_{pj},\forall p;\\ \sum_{j=1}^{J}b_{ij}\lambda_{j}+s_{i}^{b}=b_{ij},\forall i;\sum_{j=1}^{J}\lambda_{j}=1,\lambda_{j}\geq 0,\forall j;s_{n}^{x}\geq 0,\forall n;s_{m}^{e^{+}}\geq 0,\\ s_{m}^{e^{-}}\geq 0,\forall m;s_{p}^{y}\geq 0,\forall p;s_{i}^{b}\geq 0,\forall i \end{array}\right\}$$ (1)

When model (1) achieves the optimal solution status, the RAM unified efficiency index of the *t*th year of region *j* may be converted to:

$$0\leq \theta_{U}=1-(\sum_{n=1}^{N}R_{n}^{x}s_{n}^{x*}+\sum_{m=1}^{M}R_{m}^{e}(s_{m}^{e+*}+s_{m}^{e-*})+\sum_{p=1}^{p}R_{p}^{y}s_{p}^{y*}+\sum_{i=1}^{I}R_{i}^{b}s_{i}^{b*})\leq 1$$ (2)

In the above model, *x*_*nj*_ and *y*_*pj*_ are respectively the *n*th ordinary input factor and the *p*th desirable output factor in region *j*; *λ*_*j*_ is the weight of region *j*; $R_{n}^{x}$ and $R_{p}^{y}$ are respectively the adjustment range of slack $s_{n}^{x}$ and $s_{p}^{y}$, expressed as model (3). $s_{n}^{x^{*}}$ and $s_{p}^{y^{*}}$ are respectively the input slack and the output slack in the optimal solution status. *e*_*mj*_ and *b*_*ij*_ are respectively the *m*th energy input factor and the *i*th undesirable output factor in region *j*; $s_{m}^{e^{+}}$ and $s_{m}^{e^{-}}$ are the two slacks of the *m*th energy input, where the symbols + and–indicate respectively the two projection direction of energy increase and energy decrease; $R_{m}^{e}$ and $R_{i}^{b}$ are respectively the adjustment range of the slacks $s_{m}^{e^{+}},s_{m}^{e^{-}}$ and $s_{i}^{b}$, expressed as (4). In this paper, *CO*_2_ emission is the only undesirable output, and $s_{m}^{e+*},s_{m}^{e-*}$ and $s_{i}^{b*}$ are respectively the slacks of input and output in the optimal solution status.


$$\begin{array}{c} R_{n}^{x}=\frac{1}{\left(N+P)[Max(x_{nj})-Min(x_{nj})\right]}\\ R_{p}^{y}=\frac{1}{\left(N+P)[Max(y_{pj})-Min(y_{j})\right]} \end{array}\}$$ (3)

$$\begin{array}{c} R_{m}^{e}=\frac{1}{\left(N+M+I)[Max(e_{mj})-Min(e_{mj})\right]}\\ R_{i}^{b}=\frac{1}{\left(N+M+I)[Max(b_{ij})-Min(b_{ij})\right]} \end{array}\}$$ (4)

#### 3.1.2. Indicators and data

*(1) Indicator selection and data sources*. In consideration of the characteristics of transportation infrastructure in combination with research findings in existing literature, the indicators and data sources were selected to measure the unified efficiency of economy and environment of transportation infrastructure, as shown in Table 1. Due to lack of data of Tibet, Hong Kong, Macau and Taiwan, the panel data of 30 provinces and cities other than the above from 2007 to 2018 were collected in the study.

**Table 1:** Indicators used to assess unified economic and environmental efficiency of transportation infrastructure.

Category	Indicator	Quantized value	Unit	Data source
Input indicators	Capital stock	Capital stock of the transportation industry	RMB 100 million	China Statistical Yearbook
	Labor	Number of persons employed in the transportation industry	Persons	China Statistical Yearbook
	Energy consumption	Energy consumption in the transportation industry	10,000t standard coal	China Energy Statistical Yearbook
Output indicators	Desirable output	Gross production of the transportation industry	RMB 100 million	China Statistical Yearbook
		Comprehensive converted turnover of the transportation industry	100 million ton kilometer	China Statistical Yearbook
	Undesirable output	CO2 mission of the transportation industry	10,000t	China Energy Statistical Yearbook, 2006 IPCC Guidelines for National Greenhouse Gas Inventories

(2) Indicator processing.

- Capital stock: In this paper, the perpetual inventory method was used to estimate the capital stock of the transportation industry [42]. The formula used in the calculation is: *K*_*it*_ = *I*_*it*_+(1−*δ*)*K*_*it*−1_, where *K*_*it*_ is the capital stock of the transportation industry in the *t*th year of region *i*; *I*_*it*_ is the investment in transportation in the *t*th year of region, expressed here as the investment in fixed assets in transportation; *δ* is the rate of depreciation, i.e., 9.6%. To exclude the effects of the price factor, the capital stock data here were all converted to constant-price data taking 2007 as the base period.
- Labor: the number of persons employed in the transportation industry in each region.
- Energy consumption: The total consumptions of coal, gas, diesel and natural gas in the transportation industry were converted to standard coal based on their respective conversion coefficient, to obtain the total energy consumption.
- Desirable output: This includes the gross production and the comprehensive converted turnover of the transportation industry. To exclude effects of the price factor, the gross production was adjusted to a constant-price value taking 2007 as the base period based on the index of value added of the service sector. The comprehensive converted turnover was calculated based on the conversion coefficients between turnover of passenger transportation and that of freight transportation set out in the Chinese statistical system as shown in Table 2. In consideration of data availability, data of air transportation was not considerated and waterway transportation was calculated by a converted coefficient of 1/3.
- Undesirable output: *CO*_2_ emission of the transportation industry. The bottom-up method in the IPCC (2006) guidelines was used to calculate the undesirable output based the various energy consumptions. The calculation formula is: $TC_{it}=\sum_{n=1}^{m}E_{n}F_{nit}$, where *TC*_*it*_ is the *CO*_2_ emission in the *t*th year in region *i*; *E*_*n*_ is the *CO*_2_ emission factor of the *n*th energy; *F*_*nit*_ is the consumption of the *n*th energy in the *t*th year in region *i*; *m* is the type of energy consumed.

**Table 2:** Conversion coefficients used in comprehensive concerted turnover.

Transportation mode	Railway	Road	Waterway	Air
Conversion coefficient	1	1/10	1/3 (for seats) and 1 (for sleepers)	1/13

### 3.2 Panel vector autoregression model (PVAR)

In order to find whether transportation infrastructure investment can promote industrial structure transformation and upgrade and avoid ignoring possible interactions between the two, in this paper, a PVAR model was built for empirical research. The PVAR model was first put forward by Holtz-Eakin et al. [43]. Afterward, it was revised by Pesaran and Smith, Binder et al. and Love and Zicchino et al. [44–46]. PVAR model is an extension to the Vector Auto-Regression Model (VAR model). It realizes the organic combination of panel data estimation and the traditional VAR model. The VAR model doesn’t explore the internal logical relationship between variables in advance based on economic theories, but fits and predicts the responses between variables based on the statistical properties of data. All variables in the system consider their lagged terms to effectively prevent the estimation bias caused by the endogeneity of variables. The PVAR model not only has the advantages of the VAR model, but also reduces the limitation of the VAR model on the length of time series data to a certain extent. It can accurately describe the impacts of heterogeneity of different sample units on model parameters.

#### 3.2.1 Model building

The model is as shown below:

$$y_{it}=\beta_{0}+\sum_{j=1}^{p}\beta_{j}y_{it-j}+\gamma_{i}+\theta_{t}+\varepsilon_{it}$$ (5)

In the above model, *i*, *t* and *j* are respectively region, year and lag order of the model; *y*_*it*_ is a column vector containing two column vectors, namely transportation infrastructure investment (*UE*) and industrial structure transformation and upgrade. In this paper, industrial structure transformation and upgrade was measured by the industrial structure supererogation level (*Upgrade*) and the industrial structure rationalization level (*Ration*). So, the column vector can be represented by (*UE*_*it*_, *Upgrade*_*it*_, *Ration*_*it*_)^T^; *β*_0_ is the intercept; *β*_*j*_ is the regression coefficient matrix of lagged terms of endogenous variables; *γ*_*i*_ is the region effect; *θ*_*t*_ is the time effect; *ε*_*it*_ is the disturbance term of "white noise".

#### 3.2.2 Variables and Data

1. *Transportation infrastructure investment (UE)*. In this paper, the level of transportation infrastructure investment was measured by the unified economic and environmental efficiency of transportation infrastructure with the RAM model and MAXDEA software. Relevant models, indicators and data sources have been introduced in previous content.
2. *Industrial structure supererogation level (Upgrade)*. In this paper, industrial structure supererogation and industrial structure rationalization were respectively taken as the explained variables for empirical analysis. Industrial structure supererogation measures the level of industrial structure upgrade. Existing literature mainly measures the industrial structure supererogation level with the ratio of non-agricultural-sector production to GNP, the ratio of the tertiary sector’s output value to the secondary sector’s output value, Moore structural variation index and hierarchical coefficients of industrial structure. In this paper, the ratio of the tertiary sector’s output value to the secondary sector’s output value was used to calculate industrial structure supererogation. The data used for calculation come from *China Statistical Yearbooks* and *China City Statistical Yearbooks*, which collected panel data of 30 provinces and cities in China from 2007 to 2018, except Tibet, Hong Kong, Macao and Taiwan.
3. *Industrial structure rationalization level (Ration)*. Industrial structure rationalization measures the relevance and coordination between the three sectors. Existing literature mainly measures the industrial structure rationalization level with the deviation degree of industrial structure and the Theil Index. In this paper, the Theil Index was used to calculate the industrial structure rationalization level, modelled after the methods used by Gan Chunhui et al. [47]. The specific calculation formula is: ${Ration}_{it}=\sum_{m=1}^{3}\frac{Y_{mit}}{Y_{it}}ln(\frac{Y_{mit}}{L_{mit}}/\frac{Y_{it}}{L_{it}})$. In this formula, *Y*_*mit*_ and *L*_*mit*_ respectively are the *m* sector’s yield and labor of in the *t*th year in region *i*. The data used for calculation come from *China Statistical Yearbooks* and *China City Statistical Yearbooks*, which collect panel data of 30 provinces and cities in China from 2007 to 2018, except Tibet, Hong Kong, Macao and Taiwan. In addition, considering that the industrial structure rationalization level calculated by the Theil Index is a reverse index, in order to facilitate analysis, this paper adopted the range transformation method to forward the industrial structure rationalization level.

### 3.3 Mediation model and moderated mediation model

#### 3.3.1 Model building

1. *Building of mediation model*.
$${Md}_{it}=a_{0}+a_{1}{UE}_{it-1}+a_{2}X_{it-1}+\gamma_{t}+\mu_{i}+\varepsilon_{it}$$ (6)
$${Res}_{it}=b_{0}+b_{1}{UE}_{it-1}+b_{2}X_{it-1}+\gamma_{t}+\mu_{i}+\varepsilon_{it}$$ (7)
$${Tec}_{it}=c_{0}+c_{1}{UE}_{it-1}+c_{2}X_{it-1}+\gamma_{t}+\mu_{i}+\varepsilon_{it}$$ (8)
$${Ins}_{it}=d_{0}+d_{1}{UE}_{it-1}+d_{2}{Md}_{it-1}+d_{3}{Res}_{it-1}+{d_{4}Tec}_{it-1}+d_{5}X_{it-1}+\gamma_{t}+\mu_{i}+\varepsilon_{it}$$ (9)
  In the above model, *i* and *t* respectively are the region and year; *UE* is the unified economic and environmental efficiency of transportation infrastructure; *Ins* is the level of industrial structure transformation and upgrade; *Md* is the market demand level; *Res* is the resource allocation level; *Tec* is the technological innovation level; *X* is the control variables; *γ*_*t*_ is the time fixed effect; *μ*_*i*_ is the region fixed effect; *ε*_*it*_ is the random error. To avoid reverse causality between the explained variables and explanatory variables in this model, explanatory variables and control variables were all one-term lagged in this paper. The market demand level, resource allocation level and mediation effects of the resource allocation level respectively are *a*_1_*d*_2_, *b*_1_*d*_3_ *and c*_1_*d*_4_.
2. *Building of moderated mediation model*.
$${Md}_{it}=e_{0}+e_{1}{UE}_{it-1}+e_{2}{silkroad}_{i}*{post}_{t-1}+e_{3}{UE}_{it-1}{silkroad}_{i}*{post}_{t-1}+e_{4}X_{it-1}+\gamma_{t}+\mu_{i}+\varepsilon_{it}$$ (10)
$${Res}_{it}=f_{0}+f_{1}{UE}_{it-1}+f_{2}{silkroad}_{i}*{post}_{t-1}+f_{3}{UE}_{it-1}{silkroad}_{i}*{post}_{t-1}+f_{4}X_{it-1}+\gamma_{t}+\mu_{i}+\varepsilon_{it}$$ (11)
$${Tec}_{it}=g_{0}+g_{1}{UE}_{it-1}+g_{2}{silkroad}_{i}*{post}_{t-1}+g_{3}{UE}_{it-1}{silkroad}_{i}*{post}_{t-1}+g_{4}X_{it-1}+\gamma_{t}+\mu_{i}+\varepsilon_{it}$$ (12)
$${Ins}_{it}=h_{0}+h_{1}{UE}_{it-1}+h_{2}{Md}_{it-1}+h_{3}{Res}_{it-1}+h_{4}{Tec}_{it-1}+h_{5}{silkroad}_{i}*{post}_{t-1}+h_{6}{UE}_{it-1}{silkroad}_{i}*{post}_{t-1}+h_{7}X_{it-1}+\gamma_{t}+\mu_{i}+\varepsilon_{it}$$ (13)

In this model, silkroad*post are dummy variables indicating the Belt and Road Initiative, as a moderator for the model. The meanings of the rest variables are the same with those in Formulas (6)—(9). Similarly, explanatory variables and control variables were all one-term lagged in this paper. Moderated by the Belt and Road Initiative, the moderated mediation effects of the market demand level, resource allocation level and technological innovation level respectively are $h_{2}(e_{1}+e_{3}{silkroad}_{i}*{post}_{t-1}),h_{3}(f_{1}+f_{3}{silkroad}_{i}*{post}_{t-1})$ and $h_{4}(g_{1}+g_{3}{silkroad}_{i}*{post}_{t-1})$.

To reduce multicollinearity, variables in this paper, including the unified economic and environmental efficiency of transportation infrastructure, the market demand level, resource allocation level, technological innovation level, industrial structure supererogation level and industrial structure rationalization level, were standardized by means of range standardization, then the interaction terms of the unified economic and environmental efficiency of transportation infrastructure and dummy variables indicating the Belt and Road Initiative were created.

#### 3.3.2 Variables and data

This paper collected panel data of 30 provinces and cities in China from 2007 to 2018, except Tibet, Hong Kong, Macao and Taiwan. Some of the variables have been introduced, so only the selection and data sources of the mediators, moderators, and control variables are described here.

(1) *Mediators*. Mediator 1: Market demand level (*Md*)

In this paper, the market demand was divided into domestic and foreign parts, in which the scale of domestic market demand was measured by the individual consumption level in each region, and the scale of foreign market demand was measured by the export volume of each region. Variable data come from *China Statistical Yearbooks*.

Mediator 2: Resource allocation level (*Res*). In this paper, based on the theoretical framework of resource misallocation put forward by Hsieh et al., the regional resource misallocation level was measured by the capital misallocation index (*τ*_*Ki*_) and labor misallocation index (*τ*_*Li*_) modelled after studies by Chen et al. [48, 49]. The calculation formulas of the two misallocation indices are as follows:

$$\tau_{ki}=\frac{1}{\gamma_{Ki}}-1,\tau_{Li}=\frac{1}{\gamma_{Li}}-1$$ (14)

In the above formulas, *γ*_*Ki*_ and *γ*_*Li*_ respectively are the absolute distortion coefficients of capital and labor prices in region *i*, representing the addition of capital and labor prices in the absence of distortion, reflecting absolute capital and labor costs. In the actual calculation, their relative price distortion coefficients can be used instead, reflecting relative capital and labor costs. Their specific calculation formulas are as follows:

$${\hat{\gamma }}_{ki}=\left(\frac{K_{i}}{K}\right)/\left(\frac{s_{i}\beta_{Ki}}{\beta_{K}}\right),{\hat{\gamma }}_{Li}=\left(\frac{L_{i}}{L}\right)/\left(\frac{s_{i}\beta_{Li}}{\beta_{L}}\right)$$ (15)

In the above formulas, $\frac{K_{i}}{K}$ is the proportion of capital used by region *i* to total capital; $\frac{s_{i}\beta_{Ki}}{\beta_{K}}$ is the theoretical proportion of capital used by region *i* when capital is efficiently allocated; the ratio of the two measures the level of capital misallocation in region *i*; if the ratio is bigger than 1, it indicates that the actual capital allocation for region *i* is higher than the theoretical level of effective allocation, which means capital over-allocation; conversely, if the ratio is less than 1, it indicates that the actual capital allocation for region *i* is lower than the theoretical level of effective allocation, which means capital under-allocation. *s*_*i*_ is the proportion of output in region *i* to total output; *β*_*Ki*_ is the elasticity of capital output in region *i*; $\beta_{K}=\sum_{i}^{N}s_{i}\beta_{Ki}$ is the output-weighted capital contribution. Similarly, $\frac{L_{i}}{L}$ is the proportion of labor used by region *i* to total labor; $\frac{s_{i}\beta_{Li}}{\beta_{L}}$ is the theoretical proportion of labor used by region *i* when labor is efficiently allocated; the ratio of the two measures the level of labor misallocation in region *i*; if the ratio is bigger than 1, it indicates that the actual labor allocation for region *i* is higher than the theoretical level of effective allocation, which means labor over-allocation; conversely, if the ratio is less than 1, it indicates that the actual labor allocation for region *i* is lower than the theoretical level of effective allocation, which means capital under-allocation. *β*_*Li*_ is the elasticity of labor output in region *i*; $\beta_{L}=\sum_{i}^{N}s_{i}\beta_{Li}$ is the output-weighted labor contribution. Specific capital and labor output elasticity *β*_*Ki*_ and *β*_*Li*_ need to be estimated by production functions. If we adopt the Cobb-Douglas production function with constant returns to scale:

$$Y_{it}=AK_{it}^{\beta_{Ki}}L_{it}^{{1-\beta }_{Ki}}$$ (16)

After taking natural logarithms on both sides of Eq (16) and adding time fixed effect *γ*_*t*_ and region fixed effect *μ*_*i*_ to the model, the following formula can be obtained:

$$\ln\left(Y_{it}/L_{it}\right)=lnA+\beta_{Ki}\ln\left(K_{it}/L_{it}\right)+\gamma_{t}+\mu_{i}+\varepsilon_{it}$$ (17)

In the above formula, the output variable (*Y*_*it*_) is represented by regional GDP. To eliminate the impacts from price factors, in this paper, the regional GDP index is modulated into constant price data based on 2007. Labor input (*L*_*it*_) is represented by regional employees in each region at the end of the year. Capital input (*K*_*it*_) is represented by regional fixed capital stock. In this paper, capital stock was estimated by the perpetual inventory method. The output elasticity of regional capital and labor was obtained by regression estimation of Eq (17). The output elasticity of regional capital and labor may vary due to regional differences in economic and technological level, so it may be reasonable to choose a variable coefficient panel data model with variable intercept and variable slope. Therefore, the output elasticity of regional capital and labor was estimated by the least squares dummy variable (LSDV) in this paper modelled after Bai et al. [50]. After the output elasticity of regional capital and labor was estimated, regional capital and labor misallocation indices *τ*_*Ki*_ and *τ*_*Li*_ were calculated by Eq (14) and Eq (15). There may be two cases, namely over-allocation τ<0 and under-allocation τ>0. Therefore, to make the regression direction consistent for the interpretation of interaction between variables, the capital misallocation index *τ*_*Ki*_ and labor misallocation index *τ*_*Li*_ take the absolute value in this paper modelled after Ji et al. [51]. The larger these two values are, the more serious resource misallocation is. Generally speaking, when the estimated coefficients of explanatory variables are negative, that is, when the explanatory variables and the resource misallocation index change in the opposite direction, it can help reduce resource misallocation.

Mediator 3: Technological innovation level (*Tec*). In this paper, technological innovation level was measured by technological innovation quantity and technological innovation quality. Technological innovation quantity was measured by the number of granted patents in China; technological innovation quality was measured by the proportion of the sales revenue from new products of regional high-tech industries in regional GDP. To weaken the heteroscedasticity of data, technological innovation quantity takes the natural logarithm. Data of the variables involved are from *China Statistical Yearbooks* and *China Statistical Yearbooks on Science and Technology*.

In order to facilitate the analysis of mediation effects and moderated mediation effects, the multi-dimensional variables measuring each mediator were integrated into one variable for discussion in this paper. Specifically, the entropy method was used to determine the weight of variables according to the size of information presented by the observed values of each variable. The information entropy calculated to measure dimensional variables of each mediator and their corresponding weight coefficient are shown in Table 3.

**Table 3:** Information entropy and weight of dimensional variables.

Mediator	Dimensional variables	Information entropy	Weight coefficient
Market demand level (Md)	Domestic market demand scale	0.915	0.330
	Foreign market demand scale	0.827	0.670
Resource allocation level (Res)	Capital misallocation index	0.988	0.226
	Labor misallocation index	0.958	0.774
Technological innovation level (Tec)	Technological innovation quantity	0.873	0.525
	Technological innovation quality	0.885	0.475

(2) Moderator (silkroad * post). In this paper, the Belt and Road Initiative dummy variables was tested as a moderator. Specific variables are represented by the interaction terms of grouping and staging dummy variables. silkroad_i_ is the dummy variable to judge whether region *i* is a region along the routes. If region *i* is a region along the routes, *silkroad*_*i*_ is 1, otherwise 0. *post*_*t*_ is the dummy variable to judge whether the Belt and Road Initiative has been proposed in the *t*th year. The Belt and Road Initiative was put forward in 2013, so this variable is 1 for 2013 and subsequent years, and 0 for previous years.
*(3) Control variables*. The level of economic development is represented by GDP per capita; the level of urbanization is represented by the ratio of non-agricultural population to total population in each region; the level of financial development is represented by the ratio of the balance of deposits and loans in RMB at financial institutions to GDP; the level of informatization is represented by the ratio of total telecom services to GDP; transportation convenience is represented by road freight volume per capita; human capital is represented by the average length of education as a proxy variable; the level of government expenditure is represented by the ratio of local fiscal expenditure to GDP in each region; the level of marketization is represented by the ratio of the number of people employed by private businesses and self-employed to the total number of employed people in cities and towns in each region; the level of opening-up is represented by the ratio of total import and export volume to GDP in each region; the level of foreign investment is represented by the ratio of actual utilized foreign direct investment to GDP in each region. Among the control variables, data for the level of economic development, level of informatization, level of transportation convenience, level of government expenditure, level of marketization, level of foreign investment and level of opening-up, are from *China Statistical Yearbooks*; data for the level of urbanization and human capital are from *China Population and Employment Statistics Yearbooks*; data for the level of financial development are from regional *Statistical Yearbooks*.

## 4. Results and discussion

### 4.1 Impacts of the unified economic and environmental efficiency of transportation infrastructure on industrial structure transformation and upgrade

#### 4.1.1 Estimation results by Generalized Method of Moments (GMM)

In this paper, the PVAR model was estimated by the Generalized Method of Moments (GMM). The specific estimation results are shown in Table 4. Impacts of the unified economic and environmental efficiency of transportation infrastructure one-term lagged on the current level of industrial structure supererogation are significantly positive at the 10% confidence level. Impacts of the unified economic and environmental efficiency of transportation infrastructure two-term lagged on the current level of industrial structure supererogation are not significant. The unified economic and environmental efficiency of transportation infrastructure one-term lagged has no significant positive impacts on the current level of industrial structure rationalization. Impacts of the unified economic and environmental efficiency of transportation infrastructure two-term lagged on the current level of industrial structure rationalization are significantly negative at the 10% confidence level. To sum up, improvements in the unified economic and environmental efficiency of transportation infrastructure have significant positive impacts on industrial structure supererogation in the short run, but no positive impacts in the long run, insignificant positive impacts on industrial structure rationalization in the short run, but significant negative impacts in the long run. Industrial structure transformation and upgrade are considered from two dimensions, with both short-term and long-term impacts considered. Therefore, the result is that improvements in the unified economic and environmental efficiency of transportation infrastructure are conducive to industrial structure transformation and upgrade.

**Table 4:** Parameter estimation results of the PVAR model.

Variable	*UE*	*Upgrade*	*Ration*
*UE*_*t*−1_	0.792***	0.557*	0.111
	(6.34)	(1.94)	(0.90)
*UE*_*t*−2_	-0.013	-0.019	-0.133*
	(-0.14)	(-0.13)	(-1.70)
*Upgrade*_*t*−1_	0.019	0.358***	-0.023
	(0.44)	(3.01)	(-0.35)
*Upgrade*_*t*−2_	-0.004	0.292***	-0.008
	(-0.30)	(5.35)	(-0.29)
*Ration*_*t*−1_	0.018	0.658***	0.803***
	(0.33)	(2.60)	(4.74)
*Ration*_*t*−2_	0.054**	-0.153	-0.050
	(1.98)	(-1.10)	(-0.59)

Note: The values in brackets are T values

*, ** and *** respectively represent a significance level of 10%, 5%and 1%.

#### 4.1.2 Impulse response results

**Fig 2:**
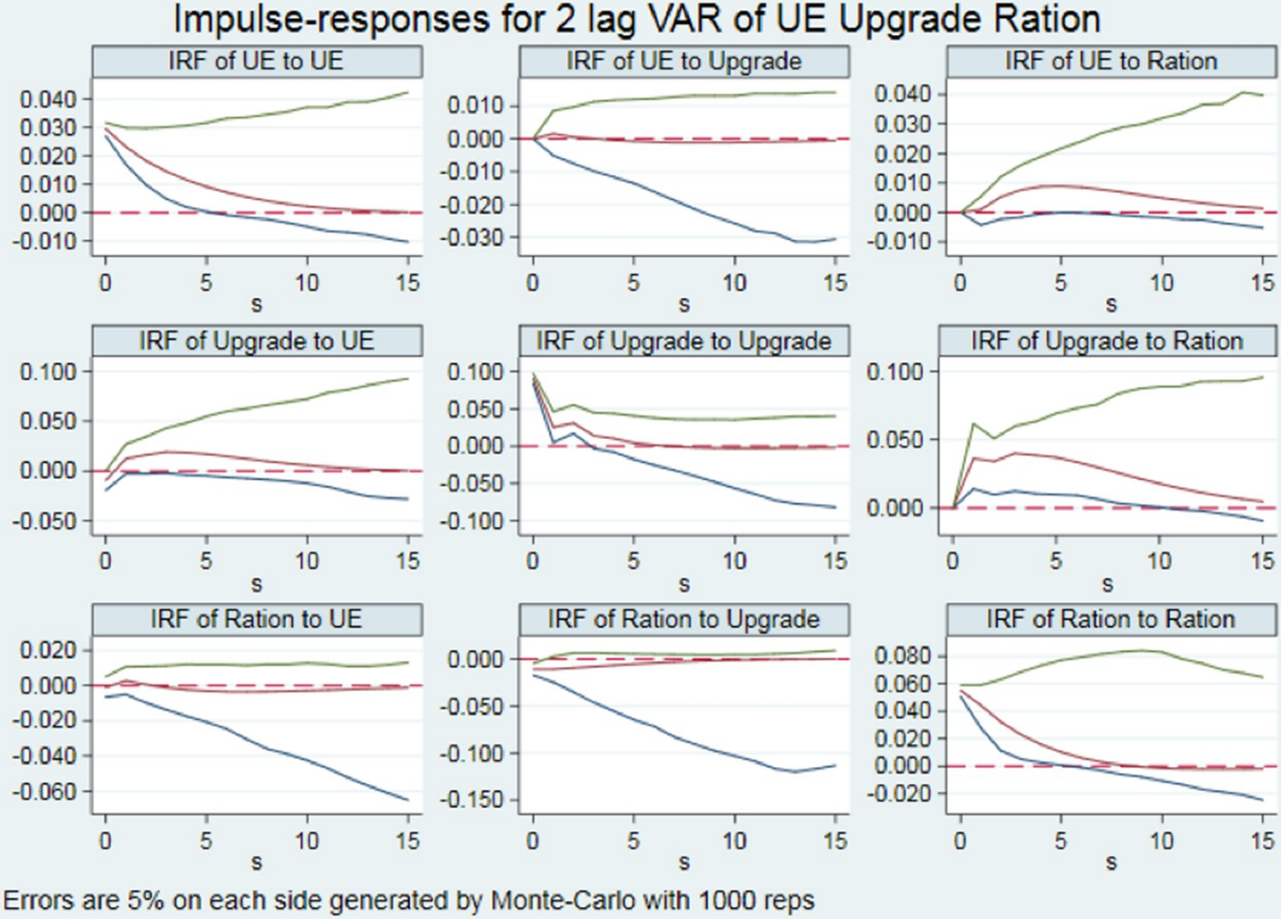
Results of impulse response.

In order to explore the long-term trend, it is necessary to analyze by impulse response functions. In this paper, based on GMM estimation, impulse response results were obtained after 1,000 Monte Carlo simulations in 95% confidence interval and 15 observation terms. Specific pulse response results are shown in Fig 2. Given one standard deviation shock, the unified economic and environmental efficiency of transportation infrastructure has temporary negative impacts on the industrial structure supererogation level in the same term; in the first term, negative impacts become positive impacts; in the third term, positive impacts reach the peak; after that, positive impacts gradually weaken and level off; it has little impacts on the industrial structure rationalization level in 15 observation terms. To sum up, from the perspective of long-term development trend, improvements in the unified economic and environmental efficiency of transportation infrastructure have positive impacts on industrial structure supererogation, but almost no impacts on industrial structure rationalization. Industrial structure transformation and upgrade are considered from two dimensions, so the result is that improvements in the unified economic and environmental efficiency of transportation infrastructure are conducive to industrial structure transformation and upgrade.

#### 4.1.3 Robustness test

**Fig 3:**
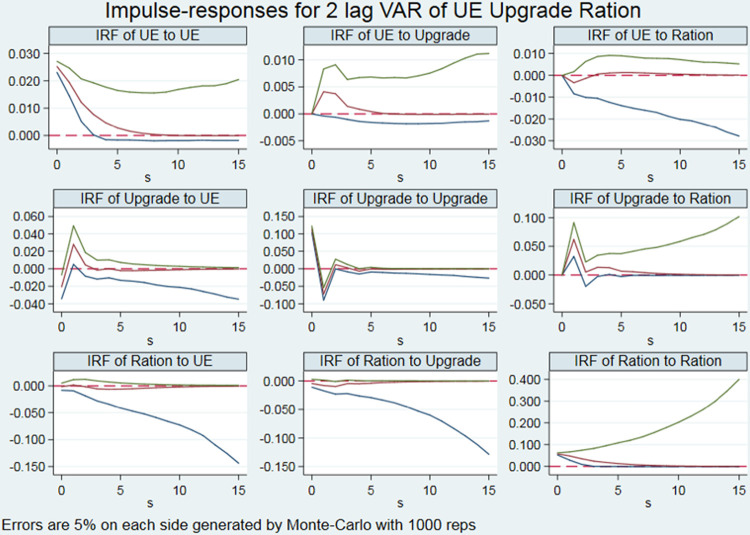
Impulse response results of robustness test.

To ensure the reliability of the above results, in this paper, a robustness test was carried out by replacing the measure indices of some variables in the models. Specifically, parameters of the two dimensional-variables measuring industrial structure transformation and upgrade were replaced. The industrial structure supererogation level was measured by using the hierarchical coefficient of industrial structure. The industrial structure rationalization level was measured by the industrial structure deviation. The range transformation method was used to forward the reverse index. The pulse response results obtained are shown in Fig 3, which shows no significant change in the overall trend of impacts. Therefore, the research conclusions of this paper are relatively robust.

### 4.2 Mechanism of the unified economic and environmental efficiency of transportation infrastructure influencing industrial structure transformation and upgrade

#### 4.2.1 Test results of mediation effect

The results of mediation model estimation by Stata 15.0 software are shown in Table 5. In column (1)—(3), with the market demand level, resource allocation level and technological innovation level as explained variables respectively, estimated coefficients of the unified economic and environmental efficiency of transportation infrastructure are significantly positive respectively at 1%, 5% and 5%. This shows that transportation infrastructure investment expands market demand, reduces resource misallocation, and promotes technological innovation.

**Table 5:** Estimation results of the mediation model.

Explained variable Explanatory variable	*Md*	*Res*	*Tec*	*Upgrade*	*Ration*
	(1)	(2)	(3)	(4)	(5)
*UE*	0.002*** (3.24)	0.001** (2.18)	0.005** (2.44)	0.128** (2.09)	0.136* (1.67)
*Md*				35.685*** (3.58)	-55.706*** (-2.80)
*Res*				-6.139 (-1.13)	2.139 (0.24)
*Tec*				5.948*** (3.85)	-11.877*** (-3.94)
Constant	0.002*** (2.73)	0.017*** (7.15)	0.003 (1.28)	1.326*** (11.75)	-0.252 (-0.55)
Control variable	Yes	Yes	Yes	Yes	Yes
Time fixed effect	Yes	Yes	Yes	Yes	Yes
Region fixed effect	Yes	Yes	Yes	Yes	Yes
Observations	360	360	360	360	360
R^2^	0.405	0.699	0.488	0.627	0.490

Note: The values in brackets are T values

*, ** and *** respectively represent a significance level of 10%, 5%and 1%.

In column (4), with the industrial structure supererogation level as the explained variable, estimated coefficients of the unified economic and environmental efficiency of transportation infrastructure are significantly positive at 5%; estimated coefficients of the market demand level and technological innovation level are significantly positive at 1%; but estimated coefficients of the resource allocation level are negative and not significant. This shows that improvements in transportation infrastructure investment, expansion of market demand and improvements in technological innovation promote industrial structure supererogation, but the resource allocation level has no positive impacts. The resource allocation level has negative impacts on industrial structure supererogation. The reasons may be as follows: the advanced development of industrial structure refers to the process of upgrading industrial structure, which is also a process in which various industrial sectors achieve the replacement of leading industrial sectors by improving output efficiency and expanding output [52]. The upgrading of industrial structure is closely related to the allocation of resources, because the upgrading process of industrial structure is not only a process of dynamic allocation of resources among industrial departments, but also a process of optimizing the efficiency of resource allocation. Specifically, the different intensity of resource allocation in each industrial sector will directly affect the output efficiency and output level of the industrial sector, and then have an impact on the upgrading of industrial structure; According to match the first clark theorem, department of resource in the process of economic development will be based on production efficiency level from low to attitude of flow, the flow from agriculture to non-agricultural other departments, and from the second industry to turn to the third industry flows, for resource reconfiguration push the departments the change of the level of output and enhance the output efficiency of each department resources, And promote the optimization and upgrading of industrial structure. The optimal allocation of resources needs to be placed in the perfect competition market as a prerequisite in order to give full play to the market competition mechanism and price mechanism on the allocation of resources. However, it is almost impossible for perfectly competitive market to exist in reality, which is due to the interference of system or policy and the existence of market failure and other factors that lead to different industrial production sectors and market players facing different resource allocation conditions. At present, our economy is still in transition and development stage. Although the market economy system reform has been carried out for a long time, the higher level market mechanism has not been fully established, which makes the market cannot play the decisive role in resource allocation fully. In addition, obstacles of institutional factors are also the main reason for resource mismatch in our country [53]. These factors mainly include industrial policy, credit restraint and tax, so resource mismatch has been prevalent in our country for a long time. Based on the dynamic mechanism of industrial structure upgrading, that is, driven by demand-side consumer structure change and supply-side factor accumulation and technological progress [54], the path of resource mismatch inhibiting industrial structure upgrading is explained from the perspective of supply and demand. Specifically, from the Angle of supply, the factor market and has not been fully developed in China, the capital market as a result of the existence of competition between local governments governance intervention by the government to a large degree, thus make use of financial credit policy intervention on the economy, the government will be to the short-term effect obvious departments (industry) orientation preferential investment and credit. In infrastructure and real estate industry, for instance, it serves to show the enthusiasm of local government, and the corresponding input and output level is much higher than the real market demand level, such a serious excess capacity and inefficient investment gains more and more low capacity utilization, and infrastructure construction of long cycle need to occupy a large amount of investment resources, As a result, the supply of strategic emerging industries and modern service industries is insufficient. In addition to capital factors, the allocation of labor factors is also affected by institutional factors, which is mainly manifested as the free flow of labor is affected by the household registration system and the extrusion effect of high housing prices, which to a certain extent leads to the shortage of high-quality labor to the high-tech industry and modern service industry. From the perspective of demand, resource allocation is affected by consumer preference. With the continuous improvement of national income level, those sectors with low elasticity of product demand seize the opportunity of rapid growth, so resources tend to flow to these sectors, thus promoting the change of industrial structure. But under the incomplete market competition, the enterprises in their production cost control, product price and commodity profit distribution due to the monopoly power will appear a certain degree of difference, the influence of factors of marginal revenue in the antitrust division will usually appear deviation, this will not only restrict the distribution of productivity in different departments, but also affect the enterprise’s technology innovation power, This will affect the level of consumer demand for scientific and technological innovation products. Therefore, from the perspective of supply and demand, it is not difficult to understand why resource mismatch inhibits the upgrading of industrial structure.

In column (5), with the industrial structure rationalization level as the explained variable, estimated coefficients of the unified economic and environmental efficiency of transportation infrastructure are significantly positive at 10%, but estimated coefficients of the market demand level and technological innovation level are both significantly negative at 1%, while estimated coefficients of the resource allocation level are positive but not significant. This shows that improvements in transportation infrastructure investment are conducive to industrial structure rationalization, but the market demand level, resource allocation level and technological innovation level have no positive impacts on industrial structure rationalization. The reasons for the negative impacts of the market demand level may be as follows: since the reform and opening up, China has mainly implemented the export-oriented economy development strategy and is highly dependent on foreign trade, which reflects that foreign trade plays a very important role in the development of China’s economy. Global import demand structure can sort according to the ratio of different types of products to the global import demand products, then make our foreign trade enterprises have to make adaptability adjustment through foreign trade means, which forms a reverse force situation to our industrial structure adjustment [55]. At the same time foreign global import demand structure change will make the investment strategies and implement investment behavior have a significant impact, especially the parts industry in China has higher FDI dependence, FDI in the aspect of regional flow and industry layout change will be a significant influence on the formation of these industries, but also based on the industry association effect to other related industries, Finally had effect on the development of rationalization of our industrial structure. Although China is a big trading country, so far it hasn’t become a real trading power. In the current global value chain system dominated by the European and American countries, the risks of China in international trade include not only trade gains losses but also the "middle-income trap" caused by long-term low value added, as well as the irrational industrial construction and the flow of resource factors, which limit the reasonable development of China’s industrial structure. Coupled with the profound adjustment of the global economic pattern and trade environment in the recent years, trade protectionism rises continuously, our foreign trade degree of dependency has declined somewhat, and the influence of foreign trade channel on our industrial structure is gradually weakening. The reasons why the resource allocation level does not have significant impacts may be as follows: the process of industrial structure rationalization refers to the process of making use of the existing conditions to rationalize the allocation of resource elements, realizing the overall and coordinated development among various industries, and finally achieving higher economic output. Under perfect market competition, resources flow from high to low with the level of production efficiency, and resource elements are rationally allocated according to the principle of efficiency. However, due to institutional factors and market factors, resource mismatch is widespread. Mismatch of resource, the resource is difficult to realize the free flow of between the industrial sector, the production efficiency is higher department unable to configure the transfer from low productivity sector of resources, resulting in higher efficiency and lower departments had insufficient configuration and configuration problem of excess, resources are difficult to realize the reasonable distribution in each production department. Without effective resource allocation, it will be difficult to realize the integration and coordinated development between industries, and the rational development of industrial structure will be inhibited. The reasons for the negative impacts of the technological innovation level may be as follows: One is technical innovation needs certain process, the ultimate goal realization depends on the quality and efficient innovation resources, and this is precisely our country lacks most of the traditional manufacturing industry and is not yet part of structural adjustment results were obtained in, many areas because of the lack of core technology with independent innovation is still in low-end value chain [56]; Secondly, the reason to promote the rationalization of industrial structure is the transfer of resource factors from the departments with lower productivity to those with higher productivity. Although we have achieved some technological innovation results through our efforts and the innovation ability deserves recognition, in reality, under the influence of institutional factors and market factors, Time lag occurs in the inter-departmental flow of production factors [57], which restricts the free flow and reasonable allocation of innovative resources. In particular, the current labor market is deeply influenced by institutional barriers [58], which greatly hindrances the free flow of labor factors between regions. It also further deepens the deviation of regional labor employment structure from industrial structure, which is not conducive to the rational development of industrial structure. Three is due to the level of industrial structure of regional technology innovation is put in bigger difference, the influence of the eastern region because of the open policy support and the good market environment, promote the progress of technological innovation and thus led to the rapid development of high and new technology industry and strategic emerging industries and industrial connection effect, the central region has a higher level of manufacturing, Technology innovation for promoting the development of the industrial structure, positive influence, but is still in the development of high and new technology industry will directly lead to innovation the industry value chain is difficult to effective integration between higher level development to realize the rationalization of industrial structure development, the western region of excessive dependence on traditional industries, high-tech talent shortage and innovation capital shortage problems, In addition, technological innovation mainly comes from the financial support of the government, which restricts the reasonable allocation of resources in other sectors, thus hindering the rational development of industrial structure [59].

In this paper, mediation effects were tested by Biased-Corrected Percentile Bootstrap (BCPB). The number of Bootstrap samplings was 1,000 in the test. If the confidence coefficient of the mediation effects is 90%, but the corresponding confidence interval does not include 0, then mediation effects are significant. The specific test results are shown in Table 6. Table 6(A) shows that when industrial structure transformation and upgrade is measured by industrial structure supererogation, the mediation effect of market demand level and technological development level was 0.060 and 0.037, respectively. The confidence intervals corresponding to the two effect values did not include 0, indicating that the mediation effect of the two was significant. This result shows that when the transformation and upgrading of industrial structure were measured from the dimension of industrial structure upgrading, transportation infrastructure investment can promote industrial structure transformation and upgrade by expanding market demand and technological innovation, so Assumption 1 and Assumption 3 are proved; the mediation effect of resource allocation level was -0.014, and the confidence interval corresponding to the effect value included 0, indicating that the mediation effect of resource allocation level was not significant. This result indicates that at the present stage, transportation infrastructure investment cannot promote industrial structure transformation and upgrade by reducing resource misallocation, so Assumption 2 is not true. The reasons for the contradiction between the reality and the theoretical analysis have been explained above. Table 6(B) shows that when industrial structure transformation and upgrade was measured by industrial structure rationalization, the mediation effect of market demand level, resource allocation level and technology development level was -0.114, -0.001 and -0.085, respectively. The confidence interval corresponding to the effect values of market demand level and technology development level did not include 0, indicating that the mediation effect of the two was significant. The confidence interval corresponding to the effect value of resource allocation level included 0, indicating that the mediation effect of resource allocation level was not significant. This result shows that when the industrial structure transformation and upgrade was measured from the dimension of industrial structure rationalization, transportation infrastructure investment cannot promote industrial structure transformation and upgrade by expanding market demand, increasing technological innovation, and reducing resource misallocation, so Assumption 1, Assumption 2 and Assumption 3 are not true. To sum up, according to theoretical analysis, transportation infrastructure investment can promote the industrial structure transformation and upgrade by expanding market demand, improving resource misallocation and technological innovation. However, due to some differences between theory and reality, empirical analysis in this paper found that in the current stage, transportation infrastructure investment can promote industrial structure supererogation by expanding market demand and technological innovation.

**Table 6:** (a): Significance test of mediation effects. (b): Significance test of mediation effects.

	Estimated value	Standard error	Z-statistic	P value	90% confidence interval	
					Lower limit	Upper limit
(a)						
*Md* mediation effects *a*_1_*d*_2_	0.060	0.030	1.99	0.046	0.011	0.110
*Res* mediation effects *b*_1_*d*_3_	-0.014	0.012	-1.14	0.225	-0.034	0.006
*Tec* mediation effects *c*_1_*d*_4_	0.037	0.020	1.81	0.071	0.003	0.071
(b)						
*Md* mediation effects *a*_1_*d*_2_	-0.114	0.054	-2.08	0.037	-0.203	-0.024
*Res* mediation effects *b*_1_*d*_3_	-0.001	0.013	-0.09	0.928	-0.022	0.020
*Tec* mediation effects *c*_1_*d*_4_	-0.085	0.042	-2.01	0.045	-0.155	-0.015

#### 4.2.2 Test results of moderated mediation effect

In view of the above mediation effect test results, only the significance of moderated mediation effects was discussed when the industrial structure supererogation level was the explained variable. Specifically, moderated mediation effects were tested by Biased-Corrected Percentile Bootstrap (BCPB). The number of Bootstrap samplings was 1,000 in the test. First, the moderating effects of the Belt and Road Initiative are tested. The specific test results are shown in Table 7. Table 7 shows that the confidence interval of *h*_2_*e*_3_ is (0.007, 0.090), the confidence interval of *h*_3_*f*_3_ is (0.021, 0.131) and the confidence interval of *h*_4_*g*_3_ is (0.008, 0.109). According to products of coefficients, the first half of the approaches for the mediation effects of transportation infrastructure investment on industrial structure transformation and upgrade through the market demand level, resource allocation level and technological innovation level are moderated by the Belt and Road Initiative. Next, whether the Belt and Road Initiative has positive moderating effects and the significance of moderated mediation effects are tested. The specific results are shown in Table 8. Moderated by the Belt and Road Initiative, the mediation effects of transportation infrastructure investment on industrial structure transformation and upgrade through the market demand level, resource allocation level and technological innovation level respectively are $h_{2}(e_{1}+e_{3}{silkroad}_{i}*{post}_{t-1}),h_{3}(f_{1}+f_{3}{silkroad}_{i}*{post}_{t-1})$ and $h_{4}(g_{1}+g_{3}{silkroad}_{i}*{post}_{t-1})$. The grouping conditions of moderators are set according to the mean of moderators plus or minus one standard deviation, so as to test the mediation effects of independent variables on dependent variables through mediators at different levels. Table 8 shows that when the moderator is valued at one standard deviation below the mean, the mean, and one standard deviation above the mean, the mediation effects of the market demand level respectively are 0.012, 0.028 and 0.052, indicating that the first half of the approaches for the mediation effects of transportation infrastructure investment on industrial structure transformation and upgrade through market demand level are positively moderated by the Belt and Road Initiative; the confidence intervals for the above three mediation effects of the market demand level respectively are (-0.008, 0.031), (0.009, 0.047) and (0.006, 0.099), indicating that with the progress of the Belt and Road Initiative, the mediation effects of transportation infrastructure investment on industrial structure transformation and upgrade through expanding market demand are increased significantly, so Assumption 4a is proved. Similarly, with the advancement of the Belt and Road Initiative, the mediation effects of transportation infrastructure investment on industrial structure transformation and upgrade by improving resource allocation and promoting technological innovation are increased significantly, so Assumption 4b and Assumption 4c are proved.

**Table 7:** Significance test of moderated mediation effects.

Products of coefficients	Estimated value	Standard error	Z-statistic	P value	90% confidence interval	
					Lower limit	Upper limit
*h*_2_ *e*_3_	0.048	0.025	1.92	0.055	0.007	0.090
*h*_3_ *f*_3_	0.076	0.033	2.28	0.022	0.021	0.131
*h*_4_ *g*_3_	0.059	0.031	1.92	0.055	0.008	0.109

**Table 8:** Significance test of moderated mediation effects at different moderator values.

	Moderator value	Estimated value	Standard error	Z-statistic	P value	90% confidence interval	
						Lower limit	Upper limit
*Md* mediation effects $h_{2}(e_{1}+e_{3}{silkroad}_{i}*{post}_{t-1})$	low	0.012	0.012	0.99	0.324	-0.008	0.031
	medium	0.028	0.012	2.38	0.017	0.009	0.047
	high	0.052	0.028	1.86	0.064	0.006	0.099
*Res* mediation effects $h_{3}(f_{1}+f_{3}{silkroad}_{i}*{post}_{t-1})$	low	-0.004	0.008	-0.46	0.646	-0.017	0.010
	medium	0.016	0.009	1.82	0.068	0.002	0.030
	high	0.045	0.025	1.78	0.076	0.003	0.087
*Tec* mediation effects $h_{4}(g_{1}+g_{3}{silkroad}_{i}*{post}_{t-1})$	low	-0.003	0.012	-0.22	0.823	-0.023	0.017
	medium	0.020	0.011	1.79	0.073	0.002	0.039
	high	0.054	0.025	2.18	0.029	0.013	0.095

Note: The low, medium and high values of moderators respectively are one standard deviation below the mean, the mean, and one standard deviation above the mean.

#### 4.2.3 Robustness test

The mediation model and moderated mediation model set above in this paper were estimated by the seemingly unrelated regression model. It is necessary to analyze the reliability of the conclusions. Given that the variables in this study have some simultaneity, robustness analysis was done by the simultaneous equation model in this paper.

*(1) Test of mediation effects*. A robustness test of mediation effects and moderated mediation effects was done with Stata 15.0. First, mediation model based on simultaneous equations were estimated by 2SLS and 3SLS respectively. The specific results are shown in Table 9. Compared with Table 5, except for slight differences in estimated coefficients of variables, there is no significant changes in the sign and significance of estimated coefficients in Table 9. Next, mediation effects were tested by Biased-Corrected Percentile Bootstrap (BCPB). The specific test results are shown in Table 10. Table 10(A) and 10(B) respectively are the test results with the industrial structure supererogation level and the industrial structure rationalization level as explained variables. In Table 10(A) and 10(B), except for slight differences in the estimated mediation effects of variables, the sign and significance of estimated values are basically the same with those in Table 6(A) and 6(B). Therefore, relevant conclusions in this paper are reliable.

**Table 9:** Estimation results of the parallel multiple mediation model based on simultaneous equations.

Explained variables Explanatory variables	Eq (6)		Eq (7)		Eq (8)		Eq (9)		Eq (9)	
	*Md*		*Res*		*Tec*		*Upgrade*		*Ration*	
*UE*	0.002***	0.002***	0.001**	0.001**	0.005**	0.006***	0.128**	0.135**	0.136*	0.135*
	(0.001)	(0.002)	(0.029)	(0.038)	(0.015)	(0.006)	(0.045)	(0.031)	(0.096)	(0.090)
*Md*							35.685***	31.151***	-55.706***	-58.415***
							(0.001)	(0.003)	(0.005)	(0.003)
*Res*							-6.139	-10.413**	2.139	-0.861
							(0.210)	(0.030)	(0.813)	(0.922)
*Tec*							5.948***	6.678***	-11.877***	-15.027***
							(0.001)	(0.000)	(0.000)	(0.000)
Constant	0.002***	0.002***	0.017***	0.021***	0.003	0.002	1.326***	1.301***	-0.252	-0.325
	(0.006)	(0.005)	(0.000)	(0.000)	(0.202)	(0.259)	(0.000)	(0.000)	(0.586)	(0.469)
Method of estimation	2SLS	3SLS	2SLS	3SLS	2SLS	3SLS	2SLS	3SLS	2SLS	3SLS
Control variable	Yes	Yes	Yes	Yes	Yes	Yes	Yes	Yes	Yes	Yes
Time/Region fixed effect	Yes	Yes	Yes	Yes	Yes	Yes	Yes	Yes	Yes	Yes
Sample quantity	360	360	360	360	360	360	360	360	360	360
R^2^	0.405	0.405	0.699	0.686	0.488	0.478	0.627	0.624	0.490	0.485

Note: The values in brackets are P values

*, ** and *** respectively represent a significance level of 10%, 5%and 1%.

**Table 10:** (a): Mediation effect significance test for robustness test. (b): Mediation effect significance test for robustness test.

Method of estimation	Mediation effects	Estimated value	Standard error	Z-statistic	P value	90% confidence interval	
						Lower limit	Upper limit
(a)							
2SLS	*Md* mediation effects *a*_1_*d*_2_	0.072	0.032	2.27	0.023	0.020	0.124
	*Res* mediation effects *b*_1_*d*_3_	-0.009	0.009	-0.93	0.352	-0.024	0.007
	*Tec* mediation effects *c*_1_*d*_4_	0.028	0.011	2.51	0.012	0.010	0.046
3SLS	*Md* mediation effects *a*_1_*d*_2_	0.060	0.030	2.00	0.046	0.011	0.110
	*Res* mediation effects *b*_1_*d*_3_	-0.014	0.012	-1.13	0.259	-0.034	0.006
	*Tec* mediation effects *c*_1_*d*_4_	0.037	0.020	1.84	0.066	0.004	0.070
(b)							
2SLS	*Md* mediation effects *a*_1_*d*_2_	-0.112	0.054	-2.08	0.037	-0.201	-0.024
	*Res* mediation effects *b*_1_*d*_3_	0.003	0.012	0.25	0.801	-0.017	0.023
	*Tec* mediation effects *c*_1_*d*_4_	-0.059	0.035	-1.69	0.091	-0.116	-0.002
3SLS	*Md* mediation effects *a*_1_*d*_2_	-0.114	0.061	-1.87	0.061	-0.213	-0.014
	*Res* mediation effects *b*_1_*d*_3_	-0.001	0.013	-0.09	0.928	-0.022	0.020
	*Tec* mediation effects *c*_1_*d*_4_	-0.085	0.044	-1.94	0.052	-0.157	-0.013

*(2) Test of moderated mediation effects*. In view of the above mediation effect test results, only the significance of moderated mediation effects with the industrial structure supererogation level as the explained variable was discussed. Specifically, moderated mediation effects were tested by Biased-Corrected Percentile Bootstrap (BCPB). First, the moderating effects of the Belt and Road Initiative are tested. The specific test results are shown in Table 11. Next, whether the Belt and Road Initiative has positive moderating effects and the significance of moderated mediation effects are tested. The specific results are shown in Table 12. The test results show that the conclusions in this paper are reliable.

**Table 11:** Significance test of moderated mediation effects as robustness test.

Method of estimation	Coefficient product	Estimated value	Standard error	Z-statistic	P value	90% confidence interval	
						Lower limit	Upper limit
2SLS	*h*_2_ *e*_3_	0.049	0.028	1.75	0.080	0.003	0.095
	*h*_3_ *f*_3_	0.075	0.028	2.64	0.008	0.028	0.121
	*h*_4_ *g*_3_	0.062	0.036	1.72	0.085	0.003	0.121
3SLS	*h*_2_ *e*_3_	0.049	0.027	1.80	0.071	0.004	0.093
	*h*_3_ *f*_3_	0.076	0.033	2.28	0.022	0.021	0.131
	*h*_4_ *g*_3_	0.057	0.031	1.84	0.066	0.006	0.108

**Table 12:** Significance test of moderated mediation effects at different moderator values as robustness test.

Method of estimation	Mediation effects	Moderator value	Estimated value	Standard error	Z-statistic	P value	90% confidence interval	
							Lower limit	Upper limit
2SLS	*Md* mediation effects $h_{2}(e_{1}+e_{3}{silkroad}_{i}*{post}_{t-1})$	low	0.012	0.011	1.05	0.295	-0.007	0.030
		medium	0.028	0.012	2.38	0.017	0.009	0.047
		high	0.052	0.027	1.94	0.053	0.008	0.097
	*Res* mediation effects $h_{3}(f_{1}+f_{3}{silkroad}_{i}*{post}_{t-1})$	low	-0.004	0.008	-0.46	0.647	-0.017	0.010
		medium	0.016	0.009	1.82	0.068	0.002	0.030
		high	0.045	0.022	2.02	0.043	0.008	0.082
	*Tec* mediation effects $h_{4}(g_{1}+g_{3}{silkroad}_{i}*{post}_{t-1})$	low	-0.003	0.012	-0.23	0.818	-0.022	0.017
		medium	0.020	0.011	1.79	0.073	0.002	0.039
		high	0.054	0.024	2.23	0.026	0.014	0.095
3SLS	*Md* mediation effects $h_{2}(e_{1}+e_{3}{silkroad}_{i}*{post}_{t-1})$	low	0.012	0.012	1.00	0.317	-0.008	0.031
		medium	0.028	0.011	2.51	0.012	0.010	0.046
		high	0.052	0.024	2.18	0.029	0.013	0.092
	*Res* mediation effects $h_{3}(f_{1}+f_{3}{silkroad}_{i}*{post}_{t-1})$	low	-0.004	0.007	-0.53	0.598	-0.015	0.008
		medium	0.016	0.008	1.97	0.049	0.003	0.029
		high	0.045	0.022	2.01	0.044	0.008	0.082
	*Tec* mediation effects $h_{4}(g_{1}+g_{3}{silkroad}_{i}*{post}_{t-1})$	low	-0.003	0.013	-0.22	0.829	-0.024	0.018
		medium	0.020	0.010	2.00	0.046	0.004	0.037
		high	0.054	0.024	2.24	0.025	0.014	0.094

Note: The low, medium and high values of moderators respectively are one standard deviation below the mean, the mean, and one standard deviation above the mean.

## 5. Conclusions

The major goal of achieving high-quality economic development is to build a modern industrial system and focus on industrial restructuring. Transportation infrastructure plays an important supporting and leading role in a modern economic system, but the impacts of development based on transportation infrastructure on different industries vary greatly, so it is necessary to find out whether transportation infrastructure investment has positive impacts on the industrial restructuring. Therefore, with China as the study object, this paper evaluates the impacts of transportation infrastructure investment on industrial structure transformation and upgrade, and explores its possible mechanism. The results show that, first, industrial structure transformation and upgrade are considered from two dimensions, with both short-term and long-term impacts considered, so improvements in the unified economic and environmental efficiency of transportation infrastructure are conducive to industrial structure transformation and upgrade. Specifically, improvements in the unified economic and environmental efficiency of transportation infrastructure have significant positive impacts on industrial structure supererogation in the short run, but no positive impacts in the long run, and insignificant positive impacts on industrial structure rationalization in the short run, but significant negative impacts in the long run. Second, transportation infrastructure investment can promote industrial structure transformation and upgrade by expanding market demand and technological innovation, and this process is positively moderated by the Belt and Road Initiative. Theoretically, transportation infrastructure investment can indirectly promote industrial structure transformation and upgrade by expanding market demand, reducing resource misallocation and conducting technological innovation, but the actual situation is different from theoretical analysis.

Policy implications of the above conclusions for further promoting industrial structure transformation and upgrade with the help of sustainable development of transportation include the following:

Proactively adapt to changes in market demand, improve resource mismatch and strengthen technological innovation. First, based on the evolution and development of China’s factor endowment, we should adapt to the trend of changing foreign demand structure from passively to actively. With the continuous improvement of people’s living standards in China, domestic market demand is expanding in scale and upgrading in structure. However, due to profound adjustments to global economic landscape and trade environment in recent years, China, which had been in the low value-added link of the global value chain system for a long time, has faced increasing difficulties in expanding foreign trade channels and expanding foreign market demand. The foreign market has always had the largest demand for labor-intensive and resource-intensive products in China. Although the two kinds of products are traditional factor endowments, they play a very limited role in promoting the transformation and upgrading of industrial structure compared with capital and technology-intensive products and service trade. At present, the labor-intensive products and the resource-intensive products are facing the rising labor cost and the non-renewable exhaustion of resources caused by the decrease of demographic dividend respectively, which will make the two kinds of products lack the future competitive advantages in the market. Although the past trade cooperation inertia and environmental change technology-intensive products and services of our country have a certain negative impact on trade demand, but considering the two types of products in the global import demand increasing share of structure, based on the perspective of dynamic comparative advantage is necessary to consider China’s factor endowments give attention to two or morethings and global changes in the demand structure, This is conducive to promoting its own embedment in the high value-added links of the global value chain. On the one hand, we need to predict the trend of the evolution of the global import demand structure through the demand analysis, so as to realize the inclusive industrial development and foreign trade strategy. On the other hand need to speed up cultivating new factor endowment, rely on to optimize the institutional environment, broaden the financing channels and measures such as encouraging technology innovation, to further strengthen technology intensive industries and modern service industry in our country in the international market competitive advantages, efforts to increase both in the export of high-tech products and offshore service outsourcing high value-added links of market share, It will change from passive adaptation to active adaptation to the change of global demand structure, and will occupy the high-end link of global value chain system in the future, which will help to realize the transformation and upgrading of Chinese industrial structure, and enhance the ability of Chinese industries to resist global risks.

Second, we should reduce resource misallocation and improve resource allocation efficiency. On the one hand, we should break administrative monopoly on resources, give full play to the decisive role of the market in allocating resources, and deepen the factor market reform. In particular, promote the reform of interest rate marketization to weaken the financial credit intervention, efforts to build the financial competition in the market environment to improve the service level of financial institutions, increase financial institutions for the industry department of small and medium-sized enterprise credit support, perfect rural financial service system to achieve the construction of a multi-level system of financial services, Finally realize the free flow and effective allocation of capital elements in all directions and at all levels; Increase proportion of Labour elements in primary distribution, we will further improve the social security system, greatly increased, including vocational skills training, to improve the quality of educational finance investment in order to promote the national labor and the cultivation of high-quality workforce, take further measures to break the household registration system and suppress prices to the free flow of labor between regional restrictions. On the other hand, we should establish a national unified market and break regional barriers, break market segmentation and trade barriers caused by the competition model of local governments. Specifically, we can reduce direct involvement of local governments in the economy, strengthen infrastructure construction, perfect the laws and regulations, and establish unified market standards for the circulation of factors and products. This will help standardize the order of market competition and improve the circulation efficiency of factors and products, and finally form a national unified market which can not only meet the requirements of producers to obtain effective resource allocation but also meet the purpose of consumers to optimize the consumption structure.

Third, we should improve scientific and technological innovation systems and mechanisms, build an open innovation system, and move industries from the middle to the high end of the value chain. First, we should stimulate scientific and technological innovation through system and mechanism innovation. We should accelerate the development of a system for technological innovation in which enterprises play the leading role and the market is the guide and industry, universities and research institutes are deeply integrated. We should focus on improving the supply of policy institutions at the institutional and institutional levels, increase policy support for enterprise innovation, and encourage enterprises to truly become the main contributors to R&D investment and the application of innovation achievements. We should further improve the intellectual property system, crack down on IPR infringements, and protect enterprises’ enthusiasm for innovation. Formulate financial policies to encourage innovation, guide financial institutions to increase technological capital investment to ensure that enterprises carry out innovative research and development activities. Second, we should build an open innovation system to explore the way of enterprise collaborative innovation. A multi-level collaborative innovation system needs to be established, in which multiple innovation factors including products, brands and business models are closely related to technological innovation, so as to realize the transformation from industrial modularization to open innovation. Third, we should strengthen the cultivation of enterprises’ independent innovation capability, promote the transformation of innovation from quantity to quality when the number of innovations reaches a certain scale, so as to gradually move the industry up the value chain.

In addition to theoretical contributions, there are still some deficiencies in this paper. First, this paper lacked research on specific transportation infrastructure investment such as highways, railways, civil aviation and water transportation, thus cannot provide specific policy guidance from the perspective of different transportation infrastructures. Subsequent studies can focus on the impacts of investment in different transportation infrastructures on industrial restructuring. Next, in this paper, the impacts of transportation infrastructure investment on industrial structure transformation and upgrade were discussed from the perspective of rational flow and optimal allocation of resource factors. Further studies can be conducted from multiple perspectives to reveal the impact mechanism between the two in depth.
